# Energy and Spectrally Efficient Modulation Scheme for IoT Applications

**DOI:** 10.3390/s18124382

**Published:** 2018-12-11

**Authors:** Hany S. Hussein, Mohamed Elsayed, Mahmoud Fakhry, Usama Sayed Mohamed

**Affiliations:** 1 Electrical Engineering Department, Faculty of Engineering, King Khalid University, Abha 61411, KSA; 2 Electrical Engineering Department, Faculty of Engineering, Aswan University, Aswan 81528, Egypt; m.fakhry@aswu.edu.eg; 3 Electrical Engineering Department, Faculty of Engineering, Sohag University, Sohag 82524, Egypt; m.elsayed@eng.sohag.edu.eg; 4 Electrical Engineering Department, Faculty of Engineering, Assiut University, Assiut 71515, Egypt; usama_s_1999@yahoo.com

**Keywords:** IoT, one-bit ADC, MIMO-CEM, OSP-MSK, energy efficiency, spectral efficiency

## Abstract

Due to the Internet of Things (IoT) requirements for a high-density network with low-cost and low-power physical (PHY) layer design, the low-power budget transceiver systems have drawn momentous attention lately owing to their superior performance enhancement in both energy efficiency and hardware complexity reduction. As the power budget of the classical transceivers is envisioned by using inefficient linear power amplifiers (PAs) at the transmitter (TX) side and by applying high-resolution analog to digital converters (ADCs) at the receiver (RX) side, the transceiver architectures with low-cost PHY layer design (i.e., nonlinear PA at the TX and one-bit ADC at the RX) are mandated to cope with the vast IoT applications. Therefore, in this paper, we propose the orthogonal shaping pulses minimum shift keying (OSP-MSK) as a multiple-input multiple-output (MIMO) modulation/demodulation scheme in order to design the low-cost transceiver architectures associated with the IoT devices. The OSP-MSK fulfills a low-power budget by using constant envelope modulation (CEM) techniques at the TX side, and by applying a low-resolution one-bit ADC at the RX side. Furthermore, the OSP-MSK provides a higher spectral efficiency compared to the recently introduced MIMO-CEM with the one-bit ADC. In this context, the orthogonality between the in-phase and quadrature-phase components of the OSP are exploited to increase the number of transmitted bits per symbol (bps) without the need for extra bandwidth. The performance of the proposed scheme is investigated analytically and via Monte Carlo simulations. For the mathematical analysis, we derive closed-form expressions for assessing the average bit error rate (ABER) performance of the OSP-MSK modulation in conjunction with Rayleigh and Nakagami-m fading channels. Moreover, a closed-form expression for evaluating the power spectral density (PSD) of the proposed scheme is obtained as well. The simulation results corroborate the potency of the conducted analysis by revealing a high consistency with the obtained analytical formulas.

## 1. Introduction

Recently, the dawn of the Internet of Things (IoT) has intensified the research efforts to fulfill the escalating demand for low-power budget wireless transceiver systems. Overnight, the communication industry experienced an explosion in the number of sensors and terminals that connect mobile users to the internet [1,2]. However, low-cost, low-power, and low-bitrate sensors and terminals are mandated for the IoT technology in order to fulfill the vast IoT applications [3]. Therefore, the mobile service operators are motivated to seek novel low-cost architectures in order to design the physical (PHY) layer associated with the IoT devices [4,5].

The IoT technology is protruded today as one of the main use cases in the upcoming generations of the wireless communication networks such as the fifth generation (5G) and beyond [6]. As such, it is expected that the IoT devices will form the main center stages that represent the major elements of the 5G networks [6]. Whereupon, serving such an enormous number of devices mandates the 5G networks to deploy a growing number of antennas i.e., hundreds or even thousands, in the base station (BS) to simultaneously serve multiple terminals over the same frequency resource. Using enormous numbers of antennas is one of the most widespread communication technologies, which is commonly termed today as massive multiple-input multiple-output (MIMO) [7,8].

In the massive MIMO, equipping the BSs with an enormous number of antennas provides a worthwhile enhancement in the system capacity, however, it results in a considerable increase in both the power consumption and the hardware complexity, since, each antenna unit requires a dedicated radio frequency (RF) chain circuit [9]. Therefore, using power-consuming elements such as RF chains hinders the massive MIMO systems being beneficially used in designing the PHY layer of the IoT devices [10]. Hence, more research efforts have been relentlessly poured into finding low-cost and low-power hardware elements, which can be efficiently used to relax both the power consumption and the hardware complexity of the massive MIMO systems. At the forefront of these efforts is the use of massive MIMO with the low-resolution one-bit analog to digital converter (ADC) at the receiver (RX) side, which comes as one of the potential solutions that can countervail the consequences of using multiple RF chains [11,12,13,14,15,16,17,18,19,20]. However, harsh quantization errors resulted as a consequence of removing most of the amplitude information of the received signal due to the use of low resolution one-bit ADC with the massive MIMO at the RX side. Hence, the channel estimation process of the massive MIMO systems with the one-bit ADC should be carefully handled for more practicality.

Referring to the existing literature, the concept of using a one-bit ADC along with the massive MIMO systems was touched upon in [11], where a least squares (LS) channel estimator based upon a maximal ratio combining (MRC) and zero-forcing (ZF) detectors was introduced to overwhelm the severe quantization noise of the one-bit ADC. Jacobsson et al. [12] extended this LS estimator to higher-order constellations in order to fulfill a higher achievable data rate than that attained in [11]. The channel is estimated based upon the near maximum likelihood (nML) detector in [13] in order to support higher-order constellations, as in [12]. A linear minimum mean square error (LMMSE) channel estimation algorithm using the Bussgang decomposition was suggested in [13]. Such an estimator was applied to both flat and frequency selective fading channels, and it offered a superior enhancement over the estimator presented in [14]. Furthermore, a pilot-based channel estimator with the Bussgang decomposition and with MRC or ZF detectors was introduced in [15]. Here, the performance of the infinite resolution case could be reached using ADCs with few bits of resolution. A joint channel-and-data (JCD) estimation algorithm based upon Bayes-optimal inference was presented in [16]. This channel estimator provides a superior enhancement when it is compared to the state whereupon the channel estimation and the data detection processes are performed independently.

The use of the one-bit ADC with massive spatial MIMO (SM-MIMO) was first tackled in [17], in which an LS channel estimator was employed for multiuser detection over frequency-selective fading channels. The implications of using low-resolution ADCs on the performance of the massive MIMO when deploying orthogonal frequency division multiplexing (OFDM) was debated in [18]. Moreover, the spectral efficiency of the one-bit ADC massive MIMO systems deploying single-carrier or OFDM transmissions was analyzed in [19]. Zhang et al. [20] extended this analysis to the performance over Rician fading channels under the effect of both perfect and imperfect channel state information. All of the aforementioned studies tried to handle the problems of the massive MIMO with a one-bit ADC at the RX side, without concerning the problems at the transmitter (TX) side. These problems included but were not limited to the high power consumption and the high hardware complexity of the massive MIMO TXs.

The multiple-input multiple-output constant envelope modulation (MIMO-CEM) with the one-bit ADC [21,22,23,24,25] was recently introduced as a low-power and low-complexity wireless communication system. In such a system, constant envelope modulation techniques such as minimum shift keying (MSK) or Gaussian minimum shift keying (GMSK) are utilized to minimize the peak to average power ratio (PAPR) of its transmitted signal. Therefore, efficient nonlinear power amplifiers (PAs) (class C or class D) are used in the MIMO-CEM at the TX side. In addition to that, due to the constant envelope feature of the transmitted signal of the MIMO-CEM, a low-resolution one-bit ADC is used at the RX side to digitalize the MIMO-CEM received signal. Consequently, the MIMO-CEM fulfills a tremendous reduction in terms of cost, power consumption, and hardware complexity. However, the MIMO-CEM with the one-bit ADC suffers from harsh quantization errors, which in turn add more challenges to the channel estimation process [21]. The channel estimation problem of the MIMO-CEM was firstly touched upon in [22], where the channel is adaptively estimated by using the preamble transmission of code division multiplexing (CDM). This CDM adaptive estimator was extended to a decision-directed channel estimator (DDCE) in order to track the fluctuation of the channel [23]. Moreover, in [24], adaptive filtering and correlation estimation were applied to estimate the MIMO-CEM channel.

The problem of the low spectral efficiency of the MIMO-CEM with the one-bit ADC was implicitly introduced in [25]. This problem without a doubt impedes the MIMO-CEM from fulfilling the high achievable data rates of the massive MIMO systems. Therefore, innovative ways should be sought to boost up the spectral efficiency of the MIMO-CEM while retaining all of its inherent advantages. To the best of the authors’ knowledge, heretofore, there have been no systematic studies in the literature concerning the spectral efficiency problem of the MIMO-CEM with the one-bit ADC until the authors proposed the MIMO orthogonal shaping pulses minimum shift keying (OSP-MSK) modulation/demodulation scheme [26]. The OSP-MSK is a low-power and low-complexity MIMO scheme that introduces a higher spectral efficiency than the MIMO-CEM by increasing the number of bits per symbol (bps) without additional bandwidth.

Ensuing the above concepts, the major contributions of this paper can be summarized as follows:

- The OSP-MSK Modulation/Demodulation Scheme: The OSP-MSK is proposed as a low-power and low-cost modulation scheme. The OSP-MSK relaxes both the power budget and the hardware complexity by using nonlinear PAs at the TX side and by deploying a low-resolution one-bit ADC at the RX side. Furthermore, the OSP-MSK achieves a higher spectral efficiency than the recent MIMO-CEM system by increasing the number of bits per symbol without additional bandwidth.
- OSP-MSK Performance Analysis: Firstly, a generic closed form expression for assessing the average bit error rate (ABER) performance of the proposed OSP-MSK modulation over an additive white Gaussian noise (AWGN) is presented thoroughly. Afterwards, this expression is used to derive closed-form expressions for the ABER performance of the OSP-MSK over the generalized Rayleigh and Nakagami-m fading channels.
- OSP-MSK Bandwidth Utilization: The bandwidth utilization of the OSP-MSK is evaluated by deriving a closed form expression of its power spectral density (PSD). This PSD is used to assess the ability of the OSP-MSK to boost the spectral efficiency compared to the MIMO-CEM system.

The rest of this paper is structured as follows. Section 2 addresses the system model of the proposed OSP-MSK modulation. The performance analysis of the OSP-MSK is driven in Section 3. The simulation results are introduced in Section 4. Finally, the paper is concluded in Section 5.

Notation: Throughout this paper, vectors are represented using bold lowercase letters and matrices are represented using bold capital letters. Complex Gaussian distributed random variables (RVs) are expressed by $CN\left(µ,\;{\sigma_{o}}^{2}\right)$, where *µ* and ${\sigma_{o}}^{2}$ denote the mean and the variance, respectively. We use $p_{\Psi }\left(\Psi \right)$ to refer to the probability density function (PDF) of a random variable Ψ. Moreover, the notations log (.), |.|, and ℜ(.) are used to indicate the natural logarithm, the magnitude, and the real part of a complex quantity, respectively. The symbol $\left(\begin{array}{c} n\\ k \end{array}\right)$ indicates the binomial coefficient of choosing n outcomes from k possibilities. The functions Q(.), erfc (.), and Γ(.) are used to indicate the *Q*-function, the complementary error function, and the gamma function, respectively. Finally, we use $2F_{1}\left(a,b;c;z\right)$ to indicate the Gaussian hypergeometric function, where *a*, *b*, and *c* are Gaussian hypergeometric function arbitrary parameters, and z is the function argument.

## 2. The OSP-MSK Modulation System Model

**Figure 1:**
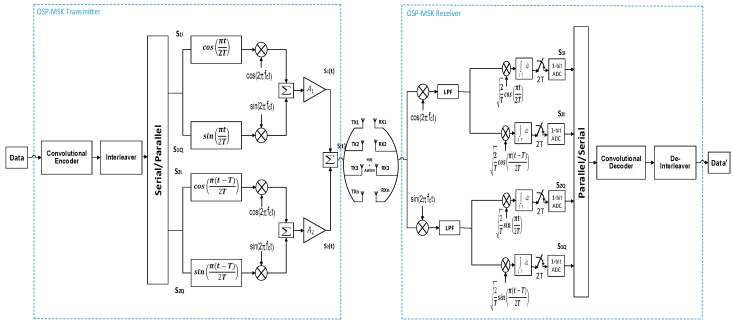
The orthogonal shaping pulses minimum shift keying (OSP-MSK) modulation system model.

Figure 1 depicts the MIMO orthogonal shaping pulses minimum shift keying (OSP-MSK) modulation system model. As shown in the figure, the input binary data are firstly encoded by using a convolutional encoder accompanied with an interleaver to make the transmitted signal of the OSP-MSK more robust against the prospective disruptions (i.e., noise, interference, multipath propagation, etc.). The convolutionally encoded data are then partitioned into two distinct sets, and each dataset is divided into in-phase components (i.e., even bits) and quadrature-phase components (i.e., odd bits). These components are then modulated using constant envelope MSK modulation, which results in a signal with a unitary peak to average power ratio (PAPR). Therefore, an energy efficient nonlinear PA is utilized to amplify each modulated dataset. Hence, a superior enhancement in the power consumption is achieved by using the OSP-MSK modulation [26].

As such, the constant envelope signal of each dataset is given by:

(1) $$S_{1}\left(t\right)=A_{1}S_{1I}\left(t\right)\cos\left(\frac{\pi t}{2T}\right)\cos\left(2\pi f_{c}t\right)+A_{1}S_{1Q}\left(t\right)\sin\left(\frac{\pi t}{2T}\right)\sin\left(2\pi f_{c}t\right),$$

which can be rewritten as follows:

(2) $$S_{1}\left(t\right)=\pm \sqrt{\frac{2E_{b}}{T}}\cos\left(\frac{\pi t}{2T}\right)\cos\left(2\pi f_{c}t\right)\pm \sqrt{\frac{2E_{b}}{T}}\sin\left(\frac{\pi t}{2T}\right)\sin\left(2\pi f_{c}t\right),$$

where $A_{1}$ is the gain of the first amplifier, $E_{b}$ is the energy per bit, T is the bit period, and $f_{c}$ is the carrier frequency. $S_{iI}\left(t\right)$ and $S_{iQ}\left(t\right)\;\mathrm{with}\;i=1,\;2$ are the in-phase and the quadrature-phase components of each MSK signal, respectively. In the same way, $S_{2}\left(t\right)$ is given by:

(3) $$S_{2}\left(t\right)=A_{2}S_{2I}\left(t\right)\cos\left(\frac{\pi \left(t-T\right)}{2T}\right)\cos\left(2\pi f_{c}t\right)+A_{2}S_{2Q}\left(t\right)\sin\left(\frac{\pi \left(t-T\right)}{2T}\right)\sin\left(2\pi f_{c}t\right),$$

(4) $$S_{2}\left(t\right)=\pm \sqrt{\frac{2E_{b}}{T}}\cos\left(\frac{\pi \left(t-T\right)}{2T}\right)\cos\left(2\pi f_{c}t\right)\pm \sqrt{\frac{2E_{b}}{T}}\sin\left(\frac{\pi \left(t-T\right)}{2T}\right)\sin\left(2\pi f_{c}t\right),$$

where $A_{2}$ denotes the gain of the second amplifier.

Therefore, the transmitted signal of the proposed OSP-MSK is expressed as follows:

(5) $$S\left(t\right)=S_{1}\left(t\right)+S_{2}\left(t\right).$$

Confusion may be arisen here that both $S_{1}\left(t\right)$ and $S_{2}\left(t\right)$ are two constant envelope signals (i.e., unitary PAPR signals), but their sum (i.e., S(t)) is not a constant envelope signal. The OSP-MSK transmitted signal S(t) is actually not a constant envelope signal, but it is a low PAPR signal (see Appendix C), because either $S_{1}\left(t\right)$ or $S_{2}\left(t\right)$ is amplified separately before it is combined with the other signal. This is in contrast to the MIMO-OFDM system, where all of the subcarriers are combined (summed) before amplifying [27]. Hence, the low PAPR property of the OSP-MSK transmitted signal enables the one-bit ADC to be beneficially used with the OSP-MSK at the RX side.

On its path, the OSP-MSK transmitted signal undergoes a fading channel, and it is corrupted by AWGN. At the RX side, a coherent demodulation is applied to the received signal, as shown in Figure 1. To extract the MSK in-phase components $S_{1I}$ and $S_{2I}$, the OSP-MSK received signal S(t) given by Equation (5) is firstly multiplied by $\cos\left(2\pi f_{c}t\right).$ Then, a low pass filter (LPF) is applied to the resulting signal in order to detect the summation of the two in-phase components $S_{I}$ as follows:

(6) $$S_{I}=\frac{1}{2}S_{1I}\cos\left(\frac{\pi t}{2T}\right)+\frac{1}{2}S_{2I}\cos\left(\frac{\pi \left(t-T\right)}{2T}\right),\;i.e.,$$

(7) $$S_{I}=\pm \sqrt{\frac{E_{b}}{2T}}\cos\left(\frac{\pi t}{2T}\right)\pm \sqrt{\frac{E_{b}}{2T}}\cos\left(\frac{\pi \left(t-T\right)}{2T}\right).$$

Finally, the MSK in-phase components are separated by exploiting the orthogonality between the shaping pulses $\left(i.e.,\;\cos\left(\frac{\pi t}{2T}\right)\;\mathrm{and}\;\cos\left(\frac{\pi \left(t-T\right)}{2T}\right)\right).$ By multiplying Equation (6) by $\sqrt{\frac{2}{T}}\cos\left(\frac{\pi t}{2T}\right)$ and applying the integration of the result over 2T, the second term in Equation (6) will vanish. Thus, the first in-phase component $S_{1I}$ can be easily extracted by using a low resolution one-bit ADC. The same procedure is applied to $S_{2I}$, but Equation (6) is multiplied by $\sqrt{\frac{2}{T}}\cos\left(\frac{\pi \left(t-T\right)}{2T}\right)$ instead of $\sqrt{\frac{2}{T}}\cos\left(\frac{\pi t}{2T}\right)$.

In the same way, the MSK quadrature-phase components $S_{1Q}$ and $S_{2Q}$ can be extracted by multiplying the OSP-MSK received signal S(t) by $\sin\left(2\pi f_{c}t\right)$. Then, a filtering process is applied to detect the summation of the two quadrature-phase components $S_{Q}$ as follows:

(8) $$S_{Q}=\frac{1}{2}S_{1Q}\sin\left(\frac{\pi t}{2T}\right)+\frac{1}{2}S_{2Q}\sin\left(\frac{\pi \left(t-T\right)}{2T}\right),\;i.e.,$$

(9) $$S_{Q}=\pm \sqrt{\frac{E_{b}}{2T}}\sin\left(\frac{\pi t}{2T}\right)\pm \sqrt{\frac{E_{b}}{2T}}\sin\left(\frac{\pi \left(t-T\right)}{2T}\right).$$

The quadrature-phase component of each dataset is then obtained by the same orthogonality principle used above. The extracted MSK in-phase and quadrature-phase components are then parallelly to serially converted. Then, a convolutional decoder and deinterleaver are applied in pursuit of estimating the input binary data.

It should be noted here that the use of low-resolution one-bit ADCs to detect the in-phase and the quadrature-phase components at the RX side of the OSP-MSK removes most of the analog stages (e.g., automatic gain control circuit (AGC), analog filters, etc.). Moreover, the use of the coherent demodulation along with the one-bit ADCs at the RX side of the OSP-MSK eliminates the need for the complex maximum-likelihood sequence estimation (MLSE) equalizer mandated at the RX side of the MIMO-CEM system [21,22,23,24,25]. Hence, with the OSP-MSK modulation, a worthwhile reduction in both the power consumption and the hardware complexity is achieved over the MIMO-CEM system.

## 3. OSP-MSK Performance Analysis

In this section, the analytical average bit error rate (ABER) performance of the proposed OSP-MSK modulation is derived. Furthermore, to evaluate the spectral efficiency of the OSP-MSK, its analytical power spectral density (PSD) is obtained in detail.

### 3.1. OSP-MSK ABER Perfrmance

**Figure 2:**
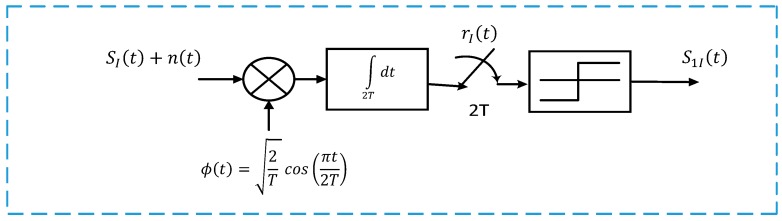
OSP-MSK correlator receiver.

The ABER performance of the OSP-MSK modulation relies mainly on the correlation receiver (i.e., four-arms correlation receiver), as shown in Figure 1. For further illustration, one arm correlation receiver of the OSP-MSK demodulator is shown in Figure 2. It is assumed here that the received signals $S_{I}\left(t\right)$ and $S_{Q}\left(t\right)$ at the inputs of the four-arms correlator receiver are corrupted by AWGN distributed as $n\left(t\right)⁓CN\left(0,\;\frac{N_{o}}{2}\right).$

Accordingly, the output of the sampler can be expressed as follows:

(10) $$r_{I}\left(2T\right)=\int_{0}^{2T}\left(S_{I}\left(t\right)+n\left(t\right)\right)\phi \left(t\right)dt,$$

(11) $$r_{I}\left(2T\right)=\underset{S_{o}}{\underbrace{\int_{0}^{2T}S_{I}\left(t\right)\phi \left(t\right)dt\;}}+\underset{n_{o}}{\underbrace{\int_{0}^{2T}n\left(t\right)\phi \left(t\right)dt}.}$$

However, in Equation (11), $S_{o}$ is a deterministic signal, but $n_{o}$ is an AWGN distributed as $n_{o}⁓CN\left(0,\frac{N_{o}}{2}\right).$ Therefore, the output of the sampler $r_{I}\left(2T\right)$ provided by Equation (11) can be rewritten as follows:

(12) $$r_{I}\left(2T\right)=\{\begin{array}{c} S_{o1}+n_{o}\;when\;“1”\;is\;transmitted\\ S_{o2}+n_{o}\;when\;“0”\;is\;transmitted \end{array},$$

and the decision threshold ξ can be expressed as follows:

(13) $$\xi =\frac{S_{o1}+S_{o2}}{2}.$$

Hence, to correctly recover “0” when $S_{o2}$ is transmitted, the term $\xi -r_{I}\left(2T\right)$ should be positive. That is:

(14) $$\frac{S_{o1}+S_{o2}}{2}-(S_{o2}+n_{o})>0.$$

Therefore, the noise $n_{o}$ should satisfy the following inequality:

(15) $$n_{o}<\frac{S_{o1}-S_{o2}}{2},$$

and the error will occur when the noise $n_{o}$ satisfies the following inequality:

(16) $$n_{o}\geq \frac{S_{o1}-S_{o2}}{2}.$$

Accordingly, the probability of error (i.e., the error in detecting “0” when $S_{o2}$ is transmitted) is given by:

(17) $$p_{e}=p\left(n_{o}\geq \frac{S_{o1}-S_{o2}}{2}\right)=\int_{\frac{S_{o1}-S_{o2}}{2}}^{\infty }\;\frac{1}{\sqrt{2\pi {\sigma_{o}}^{2}}}\;e^{-\frac{q^{2}}{2{\sigma_{o}}^{2}}}\;dq,$$

where ${\sigma_{o}}^{2}$ is the noise variance, which is equal to $\left(\frac{N_{o}}{2}\right)$. By letting $r=\frac{q}{\sqrt{2}\;\sigma_{o}}$, the probability of error can be expressed as follows:

(18) $$p_{e}=\frac{1}{\sqrt{\pi }}\int_{\frac{S_{o1}-S_{o2}}{2\sqrt{2}\;{\sigma_{o}}^{2}}}^{\infty }\;e^{-r^{2}}dr,$$

(19) $$p_{e}=\frac{1}{2}erfc\left(\frac{S_{o1}-S_{o2}}{2\sqrt{2}\;{\sigma_{o}}^{2}}\right),$$

(20) $$p_{e}=\frac{1}{2}erfc\left(\sqrt{\frac{{\left(S_{o1}-S_{o2}\right)}^{2}}{8{\sigma_{o}}^{2}}}\right).$$

However, in the proposed OSP-MSK, $S_{o}$ in Equation (11) can be obtained by letting ϕ(t) as follows:

(21) $$\phi \left(t\right)=\sqrt{\frac{2}{T}}\cos\left(\frac{\pi t}{2T}\right),$$

substituting Equation (21) in Equation (11), $S_{o}$ can be expressed as follows:

(22) $$S_{o}=\pm \sqrt{\frac{E_{b}}{T^{2}}}\overset{2T}{\underset{0}{\int }}\left[cos^{2}\left(\frac{\pi t}{2T}\right)+\cos\left(\frac{\pi t}{2T}\right)\cos\left(\frac{\pi \left(t-T\right)}{2T}\right)\right]dt=\pm \sqrt{E_{b}}.$$

Hence, $S_{o}$ will take one of two values as follows:

(23) $$S_{o}=\{\begin{array}{c} \sqrt{E_{b}}\;when\;“1”\;is\;transmitted\\ -\sqrt{E_{b}}\;when\;“0”\;is\;transmitted \end{array}.$$

Substituting Equation (23) in Equation (20), the probability of error can be expressed as follows:

(24) $$p_{e}=\frac{1}{2}erfc\left(\sqrt{\frac{E_{b}}{2{\sigma_{o}}^{2}}}\;\right)=\frac{1}{2}erfc\left(\sqrt{\frac{E_{b}}{N_{o}}}\;\right).$$

The probability of error in Equation (24) is the probability of error of the first arm correlation receiver of the OSP-MSK demodulator. Likewise, the probability of error of the second arm correlation receiver can be acquired, and it is equal to the probability of error of the first arm. However, it should be mentioned here that the second arm correlation receiver constitutes a quadrature multiplexed version of the first arm. Therefore, the probability of correctness due to the two arms can be expressed as follows:

(25) $$p_{c}={\left(1-p_{e}\right)}^{2}=1-2p_{e}+{p_{e}}^{2},$$

Hence, the probability of error due to the first two arms can be expressed as follows:

(26) $$p_{e\;1,2}=1-p_{c}=erfc\left(\sqrt{\frac{E_{b}}{N_{o}}}\;\right)-\frac{1}{4}erfc^{2}\left(\sqrt{\frac{E_{b}}{N_{o}}}\right).$$

Likewise, the probability of error due to the other two arms $p_{e\;3,4}$ can be acquired, and it equals to the probability of error due to the first two arms, $p_{e\;1,2}$. 

Lastly, the OSP-MSK demodulator can be interpreted as two independent correlation receivers, each with a probability of error of $p_{e\;1,2}$ and $p_{e\;3,4}$, respectively. Therefore, the ABER of the proposed OSP-MSK over AWGN can be expressed as follows:

(27) $$ABER_{G}=\frac{1}{2}p_{e\;1,2}+\frac{1}{2}p_{e\;3,4}=erfc\left(\sqrt{\frac{E_{b}}{N_{o}}}\;\right)-\frac{1}{4}erfc^{2}\left(\sqrt{\frac{E_{b}}{N_{o}}}\right).$$

To obtain the ABER of the proposed OSP-MSK modulation over different fading channels, the previous analysis can be expanded by averaging the ABER of AWGN provided by Equation (27) over the PDF of fading channels [28]. More specifically, when the fading is present, the received signal amplitude is attenuated by the fading amplitude *ε*, and the instantaneous signal power will be attenuated by $\varepsilon^{2}$. It is should be noted here that *ε* is a random variable (RV) with a mean square value *β* = $\bar{\varepsilon^{2}}$, and its PDF depends upon the type of fading channel. As such, the signal to noise ratio (SNR) per bit in the presence of fading can be expressed as follows:

(28) $$\Psi =\varepsilon^{2}\frac{E_{b}}{N_{o}},$$

and the average SNR per bit can be expressed as follows:

(29) $$\bar{\Psi }=\bar{\varepsilon^{2}}\frac{E_{b}}{N_{o}}=\beta \frac{E_{b}}{N_{o}}.$$

Therefore, the ABER performance of the OSP-MSK over any fading channel can be determined by integrating the channel fading effect in Equation (28) in the formula of the ABER over AWGN in Equation (27) to acquire the conditional ABER (i.e., conditioned on the channel). Then, the ABER due to the presence of fading can be expressed as follows:

(30) $$ABER_{f}=\int_{0}^{\infty }ABER_{E|H}\;p_{\Psi }\left(\Psi \right)d\Psi ,$$

where $ABER_{E|H}$ denotes the conditional ABER, and $p_{\Psi }\left(\Psi \right)$ is the PDF of the fading channel.

#### 3.1.1. Rayleigh Fading Channel

Following the aforementioned procedure, the conditional $ABER_{E|H}$ over the Rayleigh fading channel of the proposed OSP-MSK can be obtained by replacing the term $E_{b}/N_{o}$ in Equation (27) by Ψ in Equation (28) as follows:

(31) $$ABER_{E|H}=erfc\left(\sqrt{\Psi }\right)-\frac{1}{4}erfc^{2}\left(\sqrt{\Psi }\right).$$

Substituting Equation (31) into Equation (30), the ABER of the OSP-MSK over Rayleigh fading channel ($ABER_{Ray}$) can be expressed as follows:

(32) $$ABER_{Ray}=\int_{0}^{\infty }erfc\left(\sqrt{\Psi }\right)\;p_{\Psi }\left(\Psi \right)d\Psi -\frac{1}{4}\int_{0}^{\infty }erfc^{2}\left(\sqrt{\Psi }\right)\;p_{\Psi }\left(\Psi \right)d\Psi ,$$

where $p_{\Psi }\left(\Psi \right)$ is the PDF of the Rayleigh fading channel. Using the well-known PDF of the Rayleigh fading, the $ABER_{Ray}$ can be expressed as follows:

(33) $$ABER_{Ray}=\underset{I_{1}}{\underbrace{\frac{1}{\bar{\Psi }}\int_{0}^{\infty }erfc\left(\sqrt{\Psi }\right)\;e^{-\frac{\Psi }{\bar{\Psi }}}\;d\Psi \;}}-\;\underset{I_{2}}{\underbrace{\frac{1}{4\bar{\Psi }}\;\int_{0}^{\infty }\;erfc^{2}\left(\sqrt{\Psi }\right)\;e^{-\frac{\Psi }{\bar{\Psi }}}\;d\Psi }}.$$

Integral $I_{1}$ in Equation (33) is determined in detail in Appendix A, and it can be expressed as follows:

(34) $$I_{1}=1-\sqrt{\frac{\bar{\Psi }}{1+\bar{\Psi }}}.$$

However, the second integral $I_{2}$ in Equation (33) can be written in the following form:

(35) $$A=\int_{0}^{\infty }\lambda^{k_{1}-1}\;erfc^{2}\left(\sqrt{\lambda }\right)\;e^{-k_{2}\lambda }\;d\lambda ,$$

which can be easily solved as in [29] as follows:

(36) $$A=ℽ-\frac{4ℽ}{\pi }\overset{k_{1}-1}{\underset{j=0}{\sum }}\frac{{\left(k_{2}\right)}^{j}}{2j+1}2F_{1}\left(j+\frac{1}{2},j+1;j+\frac{3}{2};-\left(k_{2}+1\right)\right),$$

where ℽ is given as follows:

(37) $$ℽ=\frac{\left(k_{1}-1\right)!}{{\left(k_{2}\right)}^{k_{1}}}.$$

Hence, the second integral $I_{2}$ in Equation (33) is determined by substituting $k_{1}=1$ and $k_{2}=1/\bar{\Psi }$ in Equations (36) and (37) as follows:

(38) $$I_{2}=\frac{1}{4}-\frac{1}{\pi }*2F_{1}\left(\frac{1}{2},1;\frac{3}{2};-\left(\frac{1}{\bar{\Psi }}+1\right)\right).$$

Substituting Equations (34) and (38) into Equation (33), the ABER over the Rayleigh fading channel can be expressed as follows:

(39) $$ABER_{Ray}=\frac{3}{4}-\sqrt{\frac{\bar{\Psi }}{1+\bar{\Psi }}\;}+\frac{1}{\pi }*2F_{1}\left(\frac{1}{2},1;\frac{3}{2};-\left(\frac{1}{\bar{\Psi }}+1\right)\right).$$

#### 3.1.2. Nakagami-M Fading Channel

Using the same procedure, the ABER in the case of the Nakagami-m fading channel can be expressed as follows:

(40) $$ABER_{Nak}=\underset{I_{3}}{\underbrace{\frac{m^{m}\;}{{\bar{\Psi }}^{m}\;\Gamma \left(m\right)}\int_{0}^{\infty }erfc\left(\sqrt{\Psi \;}\right)\;\Psi^{m-1}\;e^{-\frac{m\Psi }{\bar{\Psi }}}d\Psi }}-\;\underset{I_{4}}{\underbrace{\frac{1}{4}\frac{m^{m}\;}{{\bar{\Psi }}^{m}\;\Gamma \left(m\right)}\int_{0}^{\infty }\;erfc^{2}\left(\sqrt{\Psi \;}\right)\;\Psi^{m-1}\;e^{-\frac{m\Psi }{\bar{\Psi }}}d\Psi }}.$$

Integral $I_{3}$ in Equation (40) is determined in detail in Appendix B, and it can be expressed as follows:

(41) $$I_{3}=\{\begin{array}{c} 1-U\left(\frac{\bar{\Psi }}{m}\right)\overset{m-1}{\underset{k=0}{\sum }}\left(\begin{array}{c} 2k\\ k \end{array}\right){\left(\frac{1-U^{2}\left(\frac{\bar{\Psi }}{m}\right)}{4}\right)}^{k}\;when\;m\;is\;intger\\ \frac{\sqrt{\frac{\bar{\Psi }}{m}}\;\Gamma \left(m+.5\right)}{\sqrt{\pi }\;{\left(1+\frac{\bar{\Psi }}{m}\right)}^{\frac{m+1}{2}}\Gamma \left(m+1\right)}2F_{1}\left(1,m+0.5;m+1;\frac{1}{1+\frac{\bar{\Psi }}{m}}\right)\;when\;m\;is\;non\;intger, \end{array}$$

where $U\left(\frac{\bar{\Psi }}{m}\right)$ can be expressed as follows:

(42) $$U\left(\frac{\bar{\Psi }}{m}\right)=\sqrt{\frac{\frac{\bar{\Psi }}{m}}{1+\frac{\bar{\Psi }}{m}}}.$$

Integral $I_{4}$ in Equation (40) is in the form of the integral A in Equation (35), but by substituting $k_{1}=m$ and $k_{2}=\frac{m}{\bar{\Psi }}$. Therefore, it can be determined as in Equation (36) as follows:

(43) $$I_{4}=\frac{\mathbb{Z}}{4}-\frac{\mathbb{Z}}{\pi }\overset{m-1}{\underset{j=0}{\sum }}\frac{{\left(\frac{m}{\bar{\Psi }}\right)}^{j}}{2j+1}2F_{1}\left(j+\frac{1}{2},j+1;j+\frac{3}{2};-\left(\frac{m}{\bar{\Psi }}\;+1\right)\right),$$

where ℤ is given as follows:

(44) $$\mathbb{Z}=\frac{\left(m-1\right)!}{\Gamma \left(m\right)}.$$

Substituting Equations (41) and (43) into Equation (40), the ABER over the Nakagami-m fading channel can be expressed as follows:

(45) $$ABER_{Nak}=\{\begin{array}{c} 1-U\left(\frac{\bar{\Psi }}{m}\right)\overset{m-1}{\underset{k=0}{\sum }}\left(\begin{array}{c} 2k\\ k \end{array}\right){\left(\frac{1-U^{2}\left(\frac{\bar{\Psi }}{m}\right)}{4}\right)}^{k}+\frac{\mathbb{Z}}{4}-\frac{\mathbb{Z}}{\pi }\overset{m-1}{\underset{j=0}{\sum }}\frac{{\left(\frac{m}{\bar{\Psi }}\right)}^{j}}{2j+1}2F_{1}\left(j+\frac{1}{2},j+1;j+\frac{3}{2};-\left(\frac{m}{\bar{\Psi }}+1\right)\right)\\ when\;m\;is\;intger\\ \frac{\sqrt{\frac{\bar{\Psi }}{m}}\;\Gamma \left(m+.5\right)}{\sqrt{\pi }\;{\left(1+\frac{\bar{\Psi }}{m}\right)}^{\frac{m+1}{2}}\Gamma \left(m+1\right)}2F_{1}\left(1,m+0.5;m+1;\frac{1}{1+\frac{\bar{\Psi }}{m}}\right)+\frac{\mathbb{Z}}{4}-\frac{\mathbb{Z}}{\pi }\overset{m-1}{\underset{j=0}{\sum }}\frac{{\left(\frac{m}{\bar{\Psi }}\right)}^{j}}{2j+1}2F_{1}\left(j+\frac{1}{2},j+1;j+\frac{3}{2};-\left(\frac{m}{\bar{\Psi }}+1\right)\right)\\ \;when\;m\;is\;non\;intger \end{array}$$

### 3.2. OSP-MSK Power Spectral Density

In the following, a closed form expression for the PSD of the OSP-MSK is analytically derived to evaluate the effectiveness of the proposed OSP-MSK in boosting the spectral efficiency compared to the MIMO-CEM. As depicted in Figure 1, the only main difference between the OSP-MSK and the conventional MSK is the second branch (i.e., $S_{2}\left(t\right)$) in Equation (4). Thus, the PSD of the OSP-MSK mainly relies on Equation (4). As, $\cos\left(\frac{\pi \left(t-T\right)}{2T}\right)=\sin\left(\frac{\pi t}{2T}\right)$ and $\sin\left(\frac{\pi \left(t-T\right)}{2T}\right)=\cos\left(\frac{\pi t}{2T}\right)$, then Equation (4) can be rewritten as follows:

(46) $$S_{2}\left(t\right)=\pm \sqrt{\frac{2E_{b}}{T}}\sin\left(\frac{\pi t}{2T}\right)\cos\left(2\pi f_{c}t\right)\pm \sqrt{\frac{2E_{b}}{T}}\cos\left(\frac{\pi t}{2T}\right)\sin\left(2\pi f_{c}t\right).$$

Therefore, the spectrum of $S_{1}\left(t\right)$ or $S_{2}\left(t\right)$ mainly relies on the bandwidth occupancy of the orthogonal shaping pulses $\cos\left(\frac{\pi t}{2T}\right)$ and $\sin\left(\frac{\pi t}{2T}\right).$ Accordingly, the PSD of the OSP-MSK can be obtained by deriving the PSD of the complex envelope representation of either $S_{1}\left(t\right)$ provided by Equation (2) or $S_{2}$ provided by Equation (4) [30]. As such, to obtain the PSD of $S_{1}\left(t\right)$, we can rewrite Equation (2) as follows:

(47) $$S_{1}\left(t\right)=S_{even}\left(t\right)\cos\left(2\pi f_{c}t\right)+S_{odd}\left(t\right)\sin\left(2\pi f_{c}t\right),$$

where $S_{even}\left(t\right)$ and $S_{odd}\left(t\right)$ are the in-phase and quadrature-phase components, respectively. Therefore, the complex representation of $S_{1}\left(t\right)$ can be expressed as follows:

(48) $$\tilde{S_{1}}\left(t\right)=S_{even}\left(t\right)+jS_{odd}\left(t\right).$$

However, $S_{even}\left(t\right)$ and $S_{odd}\left(t\right)$ can be expressed as follows:

(49) $$S_{even}\left(t\right)=\overset{k=\infty }{\underset{k=-\infty }{\sum }}S_{1I_{k}}\left(t\right)g\left(t-2kT\right),$$

(50) $$S_{odd}\left(t\right)=\overset{k=\infty }{\underset{k=-\infty }{\sum }}S_{1Q_{k}}\left(t\right)g\left(t-2kT\right),$$

where $S_{1I_{k}}\left(t\right),\;\mathrm{and}\;S_{1Q_{k}}\left(t\right)$ are the in-phase and quadrature-phase bits, respectively, and g(t) is the half cycle sine shaping pulse, which can be expressed as follows:

(51) $$g\left(t\right)=\{\begin{array}{c} \sqrt{\frac{2E_{b}}{T}}\sin\left(\frac{\pi t}{2T}\right)\;0\leq t\leq 2T\\ \;0\;otherwise. \end{array}$$

Since the in-phase and quadrature-phase components of the OSP-MSK signal are independent and the PSDs of $S_{even}\left(t\right)$ and $S_{odd}\left(t\right)$ are equal, the baseband PSD of $S_{1}\left(t\right)$ can be expressed as follows:

(52) $$S_{G}\left(f\right)=2\frac{{\left|G\left(f\right)\right|}^{2}}{2T},$$

where G(f) is the Fourier transform of the half-cycle shaping pulse g(t). However, the magnitude squared of G(f) can be expressed as follows:

(53) $${\left|G\left(f\right)\right|}^{2}=\frac{4E_{b}\left[1+\cos\left(4\pi fT\right)\right]}{T{\left(\frac{\pi }{2}-8\pi f^{2}T\right)}^{2}}.$$

Substituting Equation (53) into Equation (52), the baseband PSD of $S_{1}\left(t\right)$ can be expressed as follows:

(54) $$S_{G}\left(f\right)=\frac{4E_{b}\left[1+\cos\left(4\pi fT\right)\right]}{{\left(\frac{\pi }{2}-8\pi f^{2}T^{2}\right)}^{2}}.$$

Using some mathematical manipulations, the baseband PSD of $S_{1}\left(t\right)$ can be expressed as follows:

(55) $$S_{G}\left(f\right)=\frac{32E_{b}}{\pi^{2}}{\left(\frac{\cos\left(2\pi fT\right)}{1-16f^{2}T^{2}}\right)}^{2}.$$

## 4. Simulation Results

Firstly, the ABER performances of the proposed OSP-MSK over AWGN, Rayleigh fading channel, and Nakagami-m fading channel are evaluated analytically and via comprehensive Monte Carlo simulations. To compare meaningfully, the analytical formulas and the simulation results are assessed in both uncoded and coded scenarios. It should be mentioned here that the relation between the code symbol energy per $N_{o}$ and the bit energy per $N_{o}$ is expressed as follows [31]:

(56) $$\frac{E_{c}}{N_{o}}=\left(\frac{n}{k}\right)\frac{\;E_{b\;}}{N_{o}},$$

in which $\left(\frac{n}{k}\right)$ denotes the channel encoder rate.

Secondly, the ABER performance of the proposed MIMO OSP-MSK modulation is evaluated and compared to the ABER performance of the MIMO-CEM system using the traditional GMSK modulation, and by using the quadrature multiplexed Gaussian minimum shift keying (QMGMSK) modulation [32,33]. The QMGMSK modulation is a special form of the MSK modulation that boosts up the spectral efficiency of the MIMO-CEM by reducing the main lobe spectrum (i.e., the bandwidth) of the MSK modulation. The ABER comparison is performed over the Rayleigh fading channel and under different scenarios based upon the simulation parameters listed in Table 1. 

Finally, the PSD of the proposed OSP-MSK modulation is evaluated analytically and via numerical simulation and compared with the PSDs of the above-mentioned schemes in order to evaluate the spectral capabilities of the proposed OSP-MSK modulation.

### 4.1. ABER Performance

**Figure 3:**
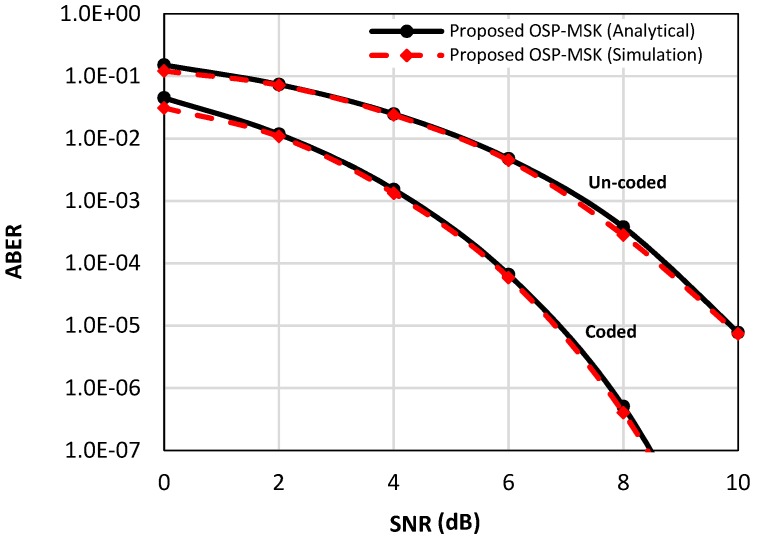
The analytical and the simulated average bit error rate (ABER) performance of the OSP-MSK modulation over additive white Gaussian noise (AWGN) for the uncoded and coded scenarios.

Figure 3 depicts the analytical ABER of the proposed OSP-MSK over AWGN in Equation (27) compared to the numerical simulation results for the uncoded and coded scenarios. As shown in Figure 3, the simulation results manifest a close match to the analytical formula over a wide range of SNR values.

**Figure 4:**
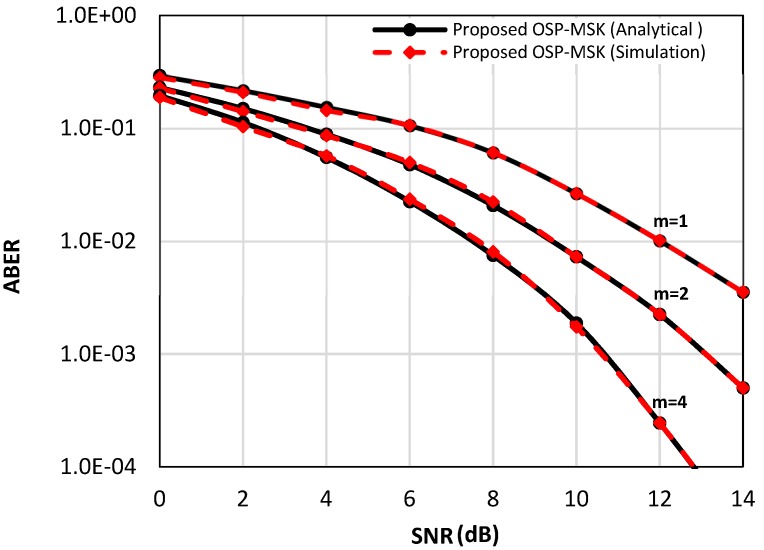
The analytical and the simulated ABER performance of the OSP-MSK modulation over the Raleigh fading channel and Nakagami-m fading channel for the uncoded scenario.

**Figure 5:**
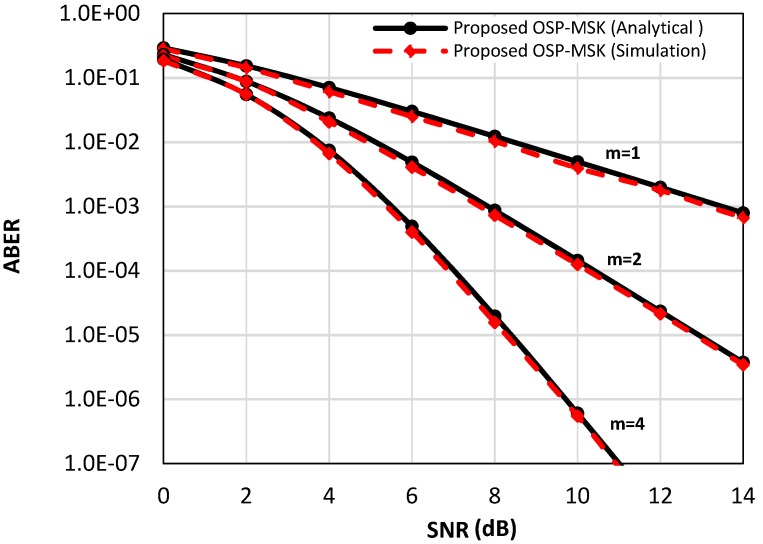
The analytical and the simulated ABER performance of the OSP-MSK modulation over the Raleigh fading channel and Nakagami-m fading channel for the coded scenario.

Figure 4 depicts the analytical ABER of the OSP-MSK modulation over the Rayleigh fading channel in Equation (39) and over the Nakagami-m fading channel in Equation (45) (e.g., *m* = two and *m* = four) compared to the numerical simulation results for the uncoded scenario. Furthermore, Figure 5 considers the coded scenario, where the convolutional encoder rate is set to ½. As depicted in Figure 4 and Figure 5, again, the simulation results are very akin to the analytical results for a wide range of SNR values.

The high consistency between the analytical formulas and the simulation results dates primarily to the absence of any mathematical assumptions in deriving the ABER of the OSP-MSK modulation. This, without a doubt, evinces the potency of the mathematical analysis conducted throughout this paper.

**Figure 6:**
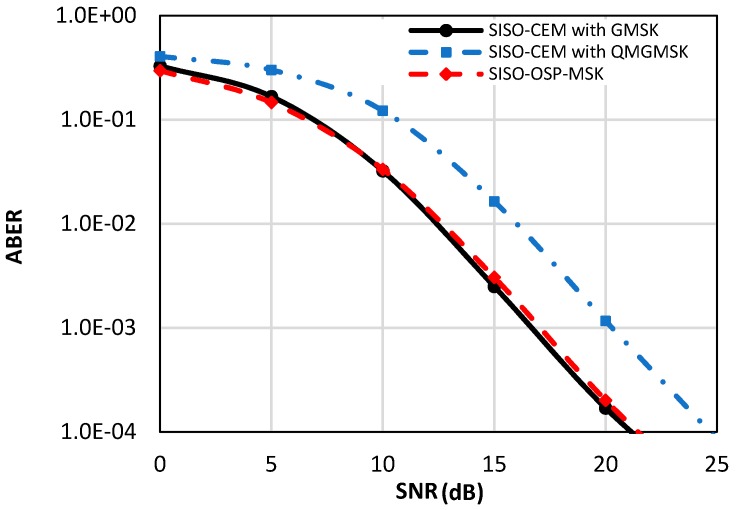
The ABER performance of the proposed single-input single-output orthogonal shaping pulses minimum shift keying (SISO-OSP-MSK) compared to the ABER of the conventional SISO-CEM system with Gaussian minimum shift keying (GMSK) and quadrature multiplexed Gaussian minimum shift keying (QMGMSK) modulation schemes [32,33].

Figure 6 depicts the ABER performance of the single-input single-output orthogonal shaping pulses minimum shift keying (SISO-OSP-MSK) modulation compared to the ABER performance of the SISO-CEM system by using GMSK and QMGMSK modulation schemes [32,33]. As depicted in Figure 6, the proposed SISO-OSP-MSK captures the same ABER performance of the conventional SISO-CEM with GMSK modulation, while the ABER performance of the SISO-CEM system is degraded by about four dB of SNR value when the QMGMSK modulation is utilized.

**Figure 7:**
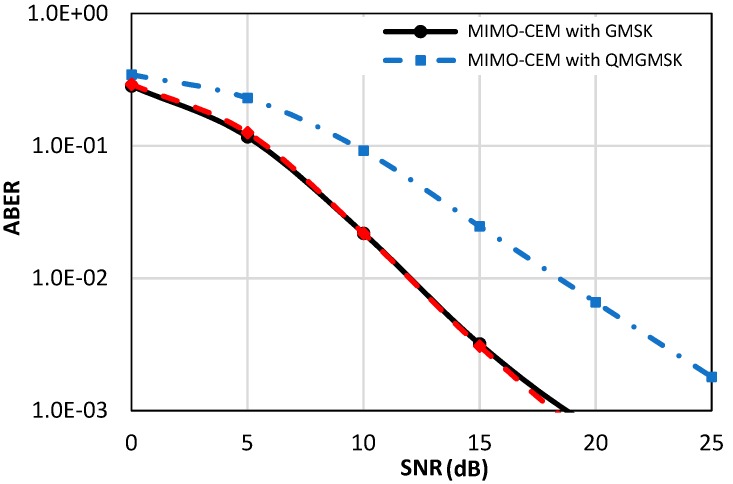
The ABER performance of the proposed MIMO-OSP-MSK compared to the ABER of the conventional multiple-input multiple-output constant envelope modulation (MIMO-CEM) system with GMSK and QMGMSK modulation schemes [32,33].

Figure 7 depicts the ABER performance of the MIMO-OSP-MSK modulation compared to the ABER performance of the MIMO-CEM system with the GMSK and QMGMSK modulation schemes, where a 2 × 2 transmit and receive antenna configuration is utilized to achieve a multiplexing gain for both of the considered schemes. As depicted in Figure 7, a comparable result is obtained in the MIMO scenario. Again, the proposed MIMO-OSP-MSK captures the same ABER performance of the conventional MIMO-CEM with GMSK modulation, while the ABER performance of the MIMO-CEM system is degraded by about five dB of SNR value when the QMGMSK modulation is utilized.

**Figure 8:**
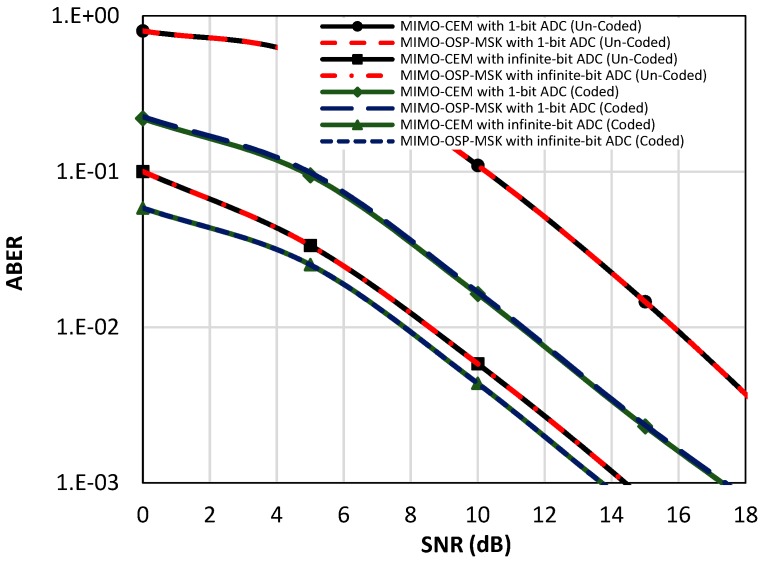
The ABER performance of the proposed MIMO-OSP-MSK compared to the ABER of the conventional MIMO-CEM system with quantization errors (i.e., one-bit ADC is utilized) and without quantization errors (i.e., infinite-bit ADC is utilized) for the uncoded and coded scenarios.

In Figure 8, the ABER performance of the MIMO-OSP-MSK modulation is evaluated and compared to the ABER performance of the conventional MIMO-CEM system in the presence of the quantization errors (i.e., one-bit ADC is utilized) and in the absence of the quantization errors (i.e., infinite-bit ADC is utilized) for the uncoded and coded scenarios (e.g., soft Viterbi decoder is utilized at the RX side). It should be noted that when the infinite-bit ADC is utilized with the OSP-MSK modulation, the use of a MLSE equalizer is mandated at the RX side in order to efficiently recover the input binary data. 

As shown in Figure 8, for the uncoded scenario, about an eight-dB difference in the SNR value is observed between the one-bit and the infinite-bit cases. While, for the coded scenario, the difference is about three dB. The prior results without a doubt manifest the effect of the harsh quantization error caused by the one-bit ADC, which severely degrades the performance of the proposed scheme as the conventional massive MIMO systems [11,12]. Furthermore, the results emphasize the importance of using channel coding techniques in both the MIMO-CEM and the proposed OSP-MSK modulation. 

### 4.2. PSD Perfomance

**Figure 9:**
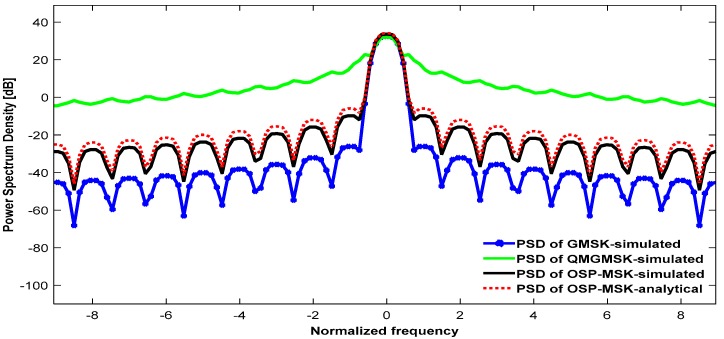
The power spectral density (PSD) of the proposed OSP-MSK compared to the PSDs of GMSK and QMGMSK.

Figure 9 depicts the analytical PSD of the OSP-MSK modulation compared to the PSD of the OSP-MSK conducted by the numerical simulation. Furthermore, the PSD of the OSP-MSK is compared with the PSDs of the different constant envelope modulation schemes (i.e., GMSK and QMGMSK) in order to corroborate the bandwidth effectiveness of the OSP-MSK modulation.

As shown in Figure 9, the spectrum of the QMGMSK [32,33] occupies about half of the main lobe spectrum of the traditional GMSK modulation. Thus, the QMGMSK approach can be used to double the spectral efficiency of the MIMO-CEM. However, unfortunately, this occurs with deterioration in the ABER performance, as shown Figure 6 and Figure 7. This dates back to the inapplicability of the QMGMSK with its non-coherent differential demodulation with the one-bit ADC in the MIMO-CEM system [32,33]. However, the spectrum of the OSP-MSK modulation occupies the same main lobe spectrum as the GMSK modulation. Therefore, the proposed OSP-MSK increases the number of transmitted bits per symbol of the conventional MSK/GMSK from two bits/symbol to four bits/symbol without the need for additional bandwidth. This undoubtedly proves the effectiveness of the proposed OSP-MSK modulation in boosting the spectral efficiency over the MIMO-CEM system.

## 5. Conclusions

In this paper, the multiple-input multiple-output orthogonal shaping pulses minimum shift keying (MIMO-OSP-MSK) has been presented as a low-cost modulation/demodulation scheme. The OSP-MSK fulfills a low-power budget by using a nonlinear PA at the TX side and by applying a low resolution one-bit ADC at the RX side. Moreover, the OSP-MSK harnesses in a new fashion the orthogonality between the shaping pulses to boost up the spectral efficiency. By means of this orthogonality, the OSP-MSK increases the number of transmitted bits per symbol of the MSK/GMSK without additional cost for extra bandwidth. Mathematical closed-form expressions for assessing the ABER performance of the OSP-MSK modulation over the AWGN channel, Rayleigh fading channel, and Nakagami-m fading channel were derived and analyzed thoroughly. In addition, a mathematical closed-form expression for the power spectral density of the proposed OSP-MSK was also derived and studied in detail. The simulation results corroborate the effectiveness of the conducted analysis by revealing a close match to the obtained analytical formulas. Furthermore, the simulation results evinced that the proposed OSP-MSK outweighs the MIMO-CEM with the one-bit ADC, in sense of the spectral efficiency and without any deterioration in the ABER performance.

## Figures and Tables

**Table 1:** Simulation Parameters. ADC: analog to digital converter, MLSE: maximum-likelihood sequence estimation.

Parameter	Value
ADC sampling rate	16 fs
Number of bits/packets	Eight bits
MLSE equalizer size	256 states
Band pass filter (BPF)	Six-order Butterworth BPF
BPF bandwidth (BW)	BW = 0.6
ADC resolution	One-bit ADC
Channel encoder	Convolutional Encoder
Channel encoder’s constraint length	Constrain length = seven
Channel encoder’s rate	rate = ½
Channel encoder’s polynomials	g_0_ = x^7^ + x^2^ + x^1^ and g_1_ = x^7^.
Channel decoder	Hard decision Viterbi decoder
Number of transmit antennas	Two antennas
Number of receive antennas	Two antennas

## Appendix A

Integral $I_{1}$ in Equation (33) can be solved by using the alternative integral expression of the *Q*-function as:

(A1) $$Q\left(x\right)=\frac{1}{\pi }\int_{0}^{\frac{\pi }{2}}e^{-\frac{x^{2}}{sin^{2}\theta }}d\theta .$$

Therefore, integral $I_{1}$ can be rewritten as follows:

(A2) $$I_{1}=\frac{2}{\bar{\Psi }\pi }\int_{0}^{\infty }\int_{0}^{\frac{\pi }{2}}e^{-\frac{\Psi }{sin^{2}\theta }}e^{-\frac{\Psi }{\bar{\Psi }}}d\theta d\Psi ,$$

(A3) $$I_{1}=\frac{2}{\bar{\Psi }\pi }\int_{0}^{\infty }\int_{0}^{\frac{\pi }{2}}e^{-\left(\frac{1}{sin^{2}\theta }+\frac{1}{\bar{\Psi }}\right)\Psi }d\theta d\Psi .$$

However, by letting B as follows:

(A4) $$B=\frac{1}{\bar{\Psi }}\int_{0}^{\frac{\pi }{2}}e^{-\left(\frac{1}{sin^{2}\theta }+\frac{1}{\bar{\Psi }}\right)\Psi }d\Psi ,$$

which can be expressed as follows:

(A5) $$B=\frac{sin^{2}\theta }{sin^{2}\theta +\bar{\Psi }}.$$

Substituting Equation (A5) into Equation (A3), the integral $I_{1}$ can be expressed as:

(A6) $$I_{1}=\frac{2}{\pi }\int_{0}^{\frac{\pi }{2}}\frac{sin^{2}\theta }{sin^{2}\theta +\bar{\Psi }}d\theta ,$$

(A7) $$I_{1}=\frac{2}{\pi }\int_{0}^{\frac{\pi }{2}}\frac{tan^{2}\left(\theta \right)}{tan^{2}\left(\theta \right)+\;\frac{\bar{\Psi }}{cos^{2}\left(\theta \right)}}d\theta ,$$

letting $u=tan\left(\theta \right)=\frac{\sin\left(\theta \right)}{\cos\left(\theta \right)}$ and using some mathematical manipulations, the integral $I_{1}$ can be expressed as follows:

(A8) $$I_{1}=\frac{2}{\pi }\int_{0}^{\frac{\pi }{2}}\frac{u^{2}}{\left(\left[1+\bar{\Psi }\right]\right)u^{2}+\bar{\Psi })\left(u^{2}+1\right)}du.$$

Using partial fraction, the integral $I_{1}$ can be rewritten as follows:

(A9) $$I_{1}=\frac{2}{\pi }\int_{0}^{\frac{\pi }{2}}\frac{-\bar{\Psi }}{\left(\left[1+\bar{\Psi }\right]\right)u^{2}+\bar{\Psi })}du+\frac{2}{\pi }\int_{0}^{\frac{\pi }{2}}\frac{1}{\left(u^{2}+1\right)}du.$$

Using formulas in [29], integral $I_{1}$ can be given as the formula in Equation (34).

## Appendix B

Integral $I_{3}$ in Equation (40) can be rewritten by using the *Q*-function alternative as follows:

(A10) $$I_{3}=\frac{2m^{m}\;}{\pi {\bar{\Psi }}^{m}\;\Gamma \left(m\right)}\int_{0}^{\infty }\int_{0}^{\frac{\pi }{2}}\Psi^{m-1}\;e^{-\left(\frac{1}{sin^{2}\theta }+\frac{m}{\bar{\Psi }}\right)\Psi }d\theta d\Psi .$$

But by letting C as follows:

(A11) $$C=\int_{0}^{\infty }\Psi^{m-1}\;e^{-\left(\frac{1}{sin^{2}\theta }+\frac{m}{\bar{\Psi }}\right)\Psi }d\Psi .$$

By using formulas in [29], the integral C can be expressed as follows:

(A12) $$C=\frac{\Gamma \left(m\right)}{{\left(\frac{1}{sin^{2}\theta }+\frac{m}{\bar{\Psi }}\right)}^{m}}=\frac{\Gamma \left(m\right)}{\frac{m^{m}}{{\bar{\Psi }}^{m}}{\left(1+\frac{1}{sin^{2}\theta }\right)}^{m}}.$$

Substituting Equation (A12) into Equation (A10), integral $I_{3}$ can be expressed as follows:

(A13) $$I_{3}=\frac{2}{\pi }\int_{0}^{\frac{\pi }{2}}{\left(1+\frac{\bar{\Psi }}{msin^{2}\theta }\right)}^{-m}d\theta .$$

The integral $I_{3}$ in Equation (A13) is definite integral whose closed-form solution can be found in [28,34] as Equation (41).

## Appendix C

The peak to average power ratio (PAPR) of the OSP-MSK transmitted signal S(t) can be expressed as follows:

(A14) $$PAPR_{S\left(t\right)}=10log_{10}\left[\frac{P_{peak}}{P_{avg}}\right]=10log_{10}\left[\frac{max\left\{{\left|S\left(t\right)\right|}^{2}\right\}}{\frac{1}{2T}\int_{0}^{2T}S^{2}\left(t\right)dt}\right],$$

where $P_{peak}$ and $P_{avg}$ are the peak and the average power of the OSP-MSK transmitted signal, respectively. However, $S^{2}\left(t\right)$ can be given as follows:

(A15) $$S^{2}\left(t\right)=a^{2}+a^{2}\sin\left(\frac{\pi t}{T}\right)\sin\left(4\pi f_{c}t\right)+\frac{a^{2}}{2}\sin\left(\frac{\pi t}{T}\right)+\frac{a^{2}}{2}\sin\left(4\pi f_{c}t\right),$$

where $a=\sqrt{\frac{2E_{b}}{T}}$. Hence, to find the maximum of $\left\{{\left|S\left(t\right)\right|}^{2}\right\}$, we let $\frac{d}{dt}\left\{S^{2}\left(t\right)\right\}=0.$ That is:

(A16) $$\begin{array}{r} \frac{d}{dt}\left\{S^{2}\left(t\right)\right\}=\left(\frac{\pi a^{2}}{T}\right)\cos\left(\frac{\pi t}{T}\right)\sin\left(4\pi f_{c}t\right)+\left(4\pi a^{2}f_{c}\right)\sin\left(\frac{\pi t}{T}\right)\cos\left(4\pi f_{c}t\right)\\ +\left(\frac{\pi a^{2}}{2T}\right)\cos\left(\frac{\pi t}{T}\right)+\left(2\pi a^{2}f_{c}\right)\sin\left(\frac{\pi t}{T}\right)\cos\left(4\pi f_{c}t\right)=0. \end{array}$$

For simplicity, let $t=\frac{T}{2}$ and $f_{c}=\frac{1}{4T}$; therefore, the right and the left-hand sides of Equation (A16) are equalized. As such, the peak power of S(t) can be obtained as follows:

(A17) $$P_{peak}=S^{2}{\left(t\right)}_{|t=\frac{T}{2},\;f_{c}=\frac{1}{4T}}=3a^{2}=\frac{6E_{b}}{T}.$$

However, the average power of S(t) is given by:

(A18) $$P_{avg}=\frac{1}{2T}\int_{0}^{2T}S^{2}\left(t\right)\mathrm{dt},$$

which can be rewritten as follows:

(A19) $$\begin{array}{c} P_{avg}=\frac{1}{2\pi }\;[\int_{0}^{2T}a^{2}dt\\ +\int_{0}^{2T}a^{2}\sin\left(\frac{\pi t}{T}\right)\sin\left(4\pi f_{c}t\right)dt\\ +\int_{0}^{2T}\frac{a^{2}}{2}\sin\left(\frac{\pi t}{T}\right)dt+\int_{0}^{2T}\frac{a^{2}}{2}\sin\left(4\pi f_{c}t\right)dt], \end{array}$$

(A20) $$\begin{array}{c} P_{avg}=\frac{1}{2\pi }\;[\int_{0}^{2T}a^{2}dt+\frac{a^{2}}{2}\int_{0}^{2T}\cos\left(\frac{\pi }{T}-4\pi f_{c}\right)tdt\\ -\frac{a^{2}}{2}\int_{0}^{2T}\cos\left(\frac{\pi }{T}+4\pi f_{c}\right)tdt\\ +\int_{0}^{2T}\frac{a^{2}}{2}\sin\left(\frac{\pi t}{T}\right)dt+\int_{0}^{2T}\frac{a^{2}}{2}\sin\left(4\pi f_{c}t\right)dt]. \end{array}$$

Therefore, the average power $P_{avg}$ in Equation (A20) is given as follows:

(A21) $$P_{avg}=a^{2}=\frac{2E_{b}}{T}.$$

Substituting equations (A17) and (A21) into Equation (A14), the PAPR of the OSP-MSK transmitted signal is given by:

(A22) $$PAPR_{S\left(t\right)}=10log_{10}\left[\frac{P_{peak}}{P_{avg}}\right]=10log_{10}\left(3\right)=4.77\mathrm{dB}.$$

The value of the PAPR of the transmitted signal S(t) provided by Equation (A22) is low compared to the high PAPR values of the OFDM system, which can be greater than 10 dB [27,35]. However, it should be mentioned here that the low PAPR property of the OSP-MSK signal doesn’t contradict with the use of nonlinear PAs at the TX side of the OSP-MSK modulation, since both $S_{1}\left(t\right)$ and $S_{2}\left(t\right)$ are constant envelope signals (i.e., unitary PAPR signals).
